# Ultrathin lensed fiber-based manual scanning optical coherence tomography needle probe for the detection of the interproximal caries

**DOI:** 10.1117/1.JBO.30.10.106001

**Published:** 2025-10-14

**Authors:** Tong Wu, Yu Zhao, Jie He, YuFei Shan, Hong Shen, Youwen Liu, YaoYao Shi, XiaoRong Gu, YuanGang Lu, Jiming Wang, ChongJun He

**Affiliations:** a Nanjing University of Aeronautics and Astronautics, College of Astronautics, Key Laboratory of Space Photoelectric Detection and Perception, Ministry of Industry and Information Technology, Nanjing, China; b The Affiliated Stomatological Hospital of Nanjing Medical University, Jiangsu Province Key Laboratory of Oral Diseases, Jiangsu Province Engineering Research Center of Stomatological Translational Medicine, Department of Endodontics, Nanjing, China; c Nanjing University of Aeronautics and Astronautics, College of Physics, Nanjing, China

**Keywords:** optical coherence tomography, needle probe, manual scanning, interproximal caries

## Abstract

**Significance:**

Interproximal caries detection is critical for effective dental treatment. We report an ultrathin lensed fiber-based manual scanning optical coherence tomography (OCT) needle probe to enables the direct imaging of the interproximal caries between two adjacent teeth.

**Aim:**

We aim to design and fabricate the ultrathin lensed fiber-based manual scanning OCT needle probe, and validate the performance of the proposed probe by applying it to the imaging of the phantom sample, the human skin tissue and the interproximal caries between two adjacent teeth.

**Approach:**

A homemade lensed fiber is packaged into a 21-gauge hypodermic needle to create a high-flexibility, ultrathin probe. A decorrelation algorithm is employed for image reconstruction based on manual scanning. The performances of the developed needle probe are experimentally measured. The probe is incorporated in a swept-source OCT system to image the phantom sample, the human skin tissue, and the interproximal caries between two adjacent teeth.

**Results:**

The working distance and focused spot diameter of the developed probe are measured to be 1.22 mm and 18.78  μm, respectively. The correctly reconstructed OCT images of the phantom, skin tissue, and the tooth tissue demonstrate the performance of the developed ultrathin lensed fiber-based manual scanning OCT needle probe. The distinct structural difference between the healthy and abnormal teeth tissue validates the efficacy of the proposed method.

**Conclusion:**

We propose an ultrathin lensed fiber-based manual scanning OCT needle probe potentially useful for the interproximal caries detection. The design, fabrication, and performances of the developed needle probe are demonstrated. We address a critical issue in the caries diagnostics and offer a promising tool for the future clinical applications.

## 1 Introduction

Interproximal caries is a highly prevalent form of tooth decay that occurs at or below the adjacent surface between two teeth. Due to the anatomical constraints, it is often difficult to clean the plaque on the adjacent surface of teeth effectively, which provides an environment conducive to cariogenic pathogens.^1^ Under the influence of the above factors, clinically diagnosed interproximal caries often approaches the pulp layer of the teeth. Therefore, diagnosis and treatment of interproximal caries are very important for the preservation of dental tissue. The traditional visual and tactile examination methods for the diagnosis of dental caries rely on human sensory identification, which cannot adequately assess the interproximal caries.^2^ The current gold standard for the dental caries diagnosis is histological sectioning and microCT. However, histological sectioning is an invasive method and requires excising the tooth tissue. To obtain high-resolution images, microCT requires higher doses of X-ray radiation.

Optical coherence tomography (OCT), a high-resolution and high-contrast optical imaging technique, has demonstrated significant efficacy in detecting caries, compared with conventional medical imaging modalities.^3^^–^^5^ To image the tooth tissue *in vivo*, an endoscopic probe should be incorporated in the OCT system. In recent years, various research groups have proposed a variety of OCT probe designs for dental diagnosis. Fujimoto et al.^6^ have successfully developed two types of miniature forward-viewing endoscopic probes for dental root canal observation, achieving minimally invasive and high-precision visual examination of the root canal system. Won et al.^7^ designed a visually adjustable OCT probe based on a MEMS scanning mirror to image the dental plaque and calculus in the inner part of incisor and molar teeth. Choi et al.^8^ designed and developed a hand-held, MEMS-based scanning mirror intraoral spectral-domain optical coherence tomography (SD-OCT) probe, enabling imaging of the hard tissues (such as enamel and dentin) of the extracted teeth. Some commercial companies have also designed and developed the probes for caries detection, such as the right-angle 2D scanning probes commercially available from Santec Inc. Although the accuracy of these mechanical scanning-based probes is high, they are still bulky in size and are unable to image the adjacent surface through the tiny crack between a front tooth and a back tooth. A research team reported an OCT-based rotary catheter probe enabling the imaging of the early dental caries through the tiny crack between a front tooth and a back tooth.^9^ However, this kind of rotary catheter probe covers only a limited fan-shaped field of view, and the focused beam becomes oblique in the marginal region, making it inappropriate for this application.

A more feasible way for the dentist to manipulate the endoscopic probe in a clinical setting is to perform manual scanning. The high degree of freedom provided by manual scanning facilitates the smooth control and guidance of the probe on the imaging target. However, it is inevitable to introduce velocity and trajectory variations, causing geometric distortions in the OCT images. Researchers have developed several methods to address the issue, such as location tracking and speckle decorrelation.^10^^–^^14^ Among them, the speckle decorrelation algorithm is particularly interesting. Ahmad et al.^10^ pioneered the speckle decorrelation algorithm for an OCT system. Liu et al.^11^ extended this work by establishing a speckle theoretical model to extract probe displacement from correlation values without experimental calibration.

In this paper, we present an ultrathin lensed fiber-based manual scanning OCT needle probe for the direct detection of interproximal caries. Its high flexibility and ultrathin outer diameter enable unprecedented access to interproximal surfaces between teeth. In the following sections, the design and principle of the manual scanning needle probe are introduced. Fabrication and testing of the probe are described. The reconstructed images of the phantom, biological tissues are illustrated, demonstrating the ability of the proposed probe in the detection of the interproximal caries.

## 2 Materials and Methods

### 2.1 SS-OCT System

**Fig. 1:**
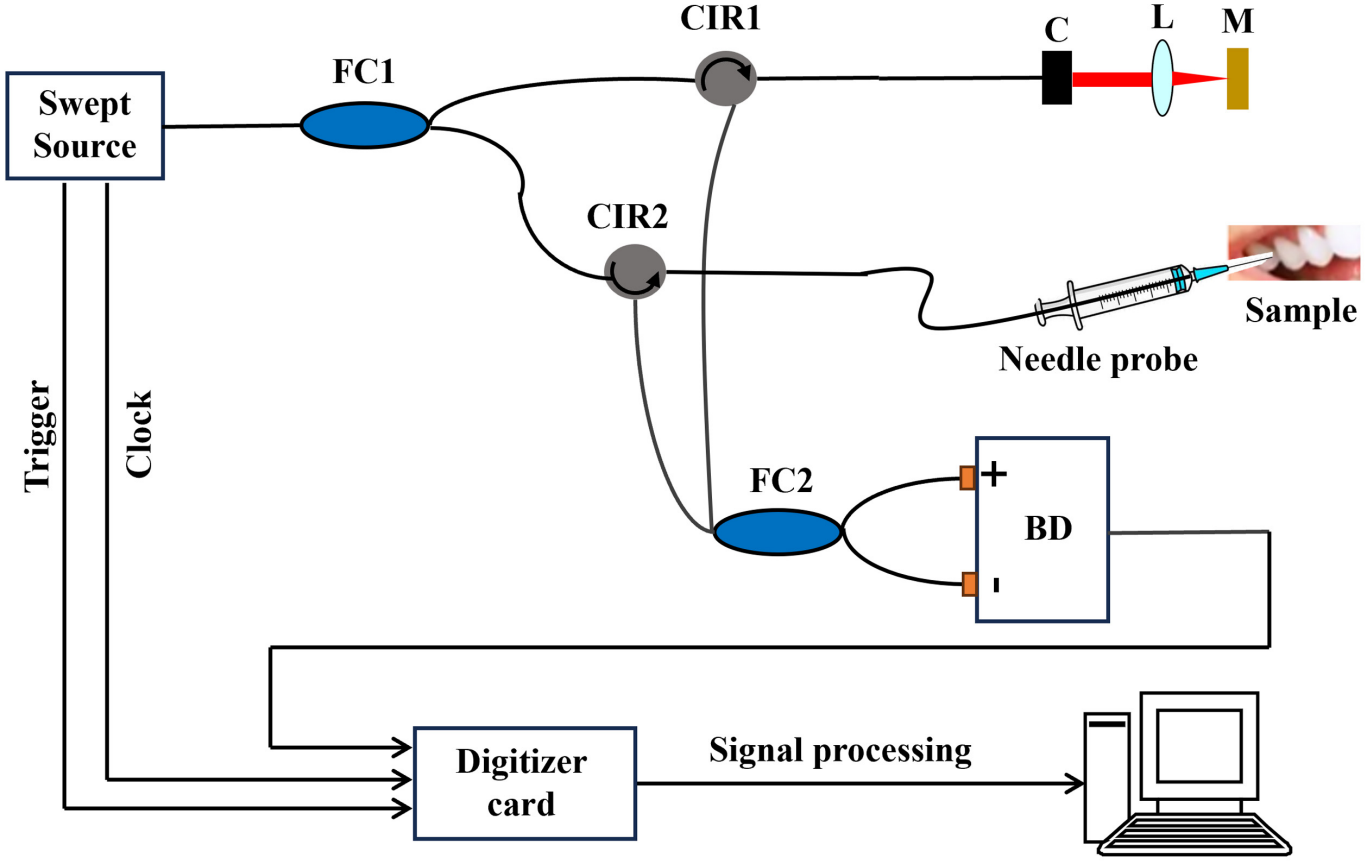
Schematic of the manual scanning OCT probe-based SS-OCT system. FC, fiber coupler; CIR, circulator; C, collimator; L, lens; M, mirror; and BD, balanced photodetector.

The schematic of the lensed fiber-based endoscopic SS-OCT system is shown in Fig. 1. The swept source (Axsun, USA) has a central wavelength of 1310 nm and a scanning frequency of 50 kHz. The light emitted from the swept source is split by a 90/10 single-mode optical fiber coupler (FC1). The 10% output port of FC1 directs light into the reference arm via a circulator (CIR1). The reference arm consists of a collimator, a focusing lens, and a mirror. The 90% output port of the FC1 directs light to the sample arm containing solely the homemade needle probe via another circulator (CIR2). The interference signal is detected by a balanced photodetector, and the electrical signal from the photodetector is digitized by the data acquisition card (ATS-9350 Alazar Tech, Canada).

### 2.2 OCT Imaging Needles

#### 2.2.1 Design of the lensed fiber-based needle probe

**Fig. 2:**
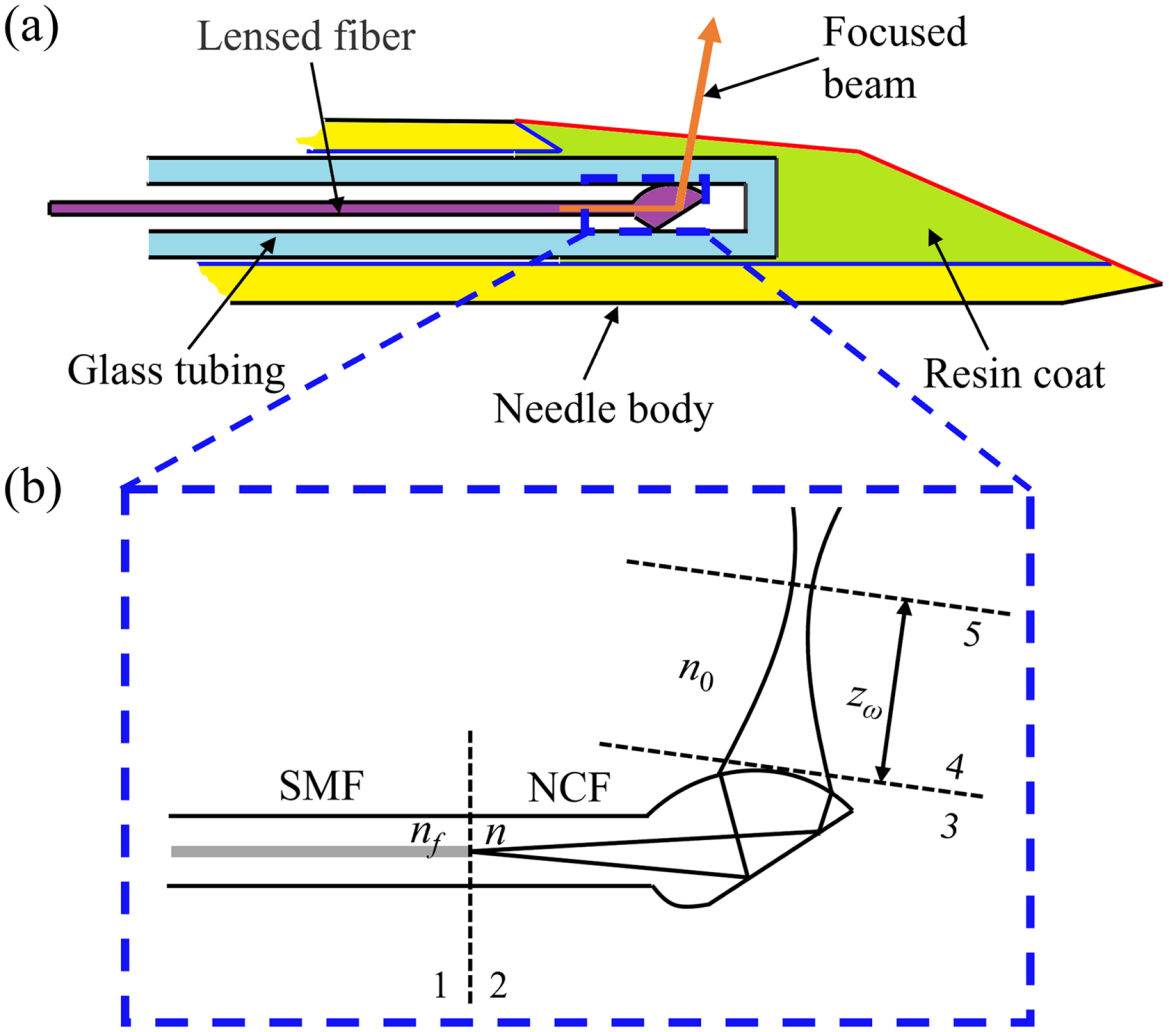
(a) Schematic of the distal end of the proposed lensed fiber-based needle probe and (b) the equivalent forward-viewing model of the probe. SMF, single-mode fiber; NCF, no-core fiber; $z_{\omega }$, working distance; n, refractive index of the NCF; $n_{0}$, refractive index of the medium; and $n_{f}$, refractive index of the SMF.

The schematic of the distal end of the proposed lensed fiber-based needle probe is presented in Fig. 2(a). The needle probe consists of lensed fiber, a glass capillary tube, an outer needle body, and a resin coating. For side-view imaging, the fiber ball lens is designed with a total internal reflection (TIR) surface to deflect the light beam. To ensure the TIR, the lensed fiber is inserted into a glass capillary tube to isolate it from the external medium. The glass tube is placed inside the needle, and the resin coating is applied to protect the probe from the outer substance.

As shown in Fig. 2(b), the lensed fiber consists of a segment of single-mode fiber (SMF), a segment of the no-core fiber (NCF), and a fiber ball lens. The NCF acts as an optical spacer between the SMF and the fiber ball lens, facilitating the beam expansion. The fiber ball lens features an angle-polished surface to deflect the incident beam and focus the outgoing beam by its curved end surface.

To determine the optimal structural parameters of the probe, ray tracing analysis based on the ABCD matrix formalism was performed. As illustrated in Fig. 2(b), the key design parameters are the length of the NCF (L) and the radius of curvature of the fiber ball lens (r). Length L is defined as the distance from planes 2 to 4.

The ABCD matrix for refraction at the interface between the SMF end facet (plane 1) and the NCF (plane 2) can be expressed as

$$M_{12}=[\begin{array}{cc} 1 & 0\\ 0 & \frac{n_{f}}{n} \end{array}],$$ (1)

where $n_{f}$ is the refractive index of the core of the SMF and n is the refractive index of NCF.

The ABCD matrix of the light propagation from the input plane of the NCF to the curved end surface of the ball lens can be expressed as

$$M_{23}=[\begin{array}{cc} 1 & L\cdot n\\ 0 & 1 \end{array}].$$ (2)

The ABCD matrix of the refraction at the curved end surface of the ball lens can be expressed as

$$M_{34}=[\begin{array}{cc} 1 & 0\\ \frac{n_{0}-n}{n_{0}r} & \frac{n}{n_{0}} \end{array}],$$ (3)

where $n_{0}$ is the refractive index of the surrounding medium.

The ABCD matrix of the light transmission from the end surface of the fiber ball lens to the focus spot can be expressed as

$$M_{45}=[\begin{array}{cc} 1 & z_{\omega }\\ 0 & 1 \end{array}],$$ (4)

where $z_{\omega }$ is the distance from the tip of the fiber ball lens to the focal plane, which is defined as the working distance of the probe.

Thus, the total transformation matrix M of the probe can be expressed as

$$M=[\begin{array}{cc} A & B\\ C & D \end{array}]=M_{45}M_{34}M_{23}M_{12}.$$ (5)

The transmission of a Gaussian beam is characterized by its complex parameter q, which is expressed as

$$\frac{1}{q}=\frac{1}{R}-\frac{i\lambda }{n_{0}\pi \omega^{2}},$$ (6)

where R is the radius of curvature of the wavefront, ω is the beam radius at a certain position, and λ is the optical wavelength. Parameters R and ω at any position can be derived from the complex parameter q.

The ABCD matrix transformation of the complex parameter from planes 1 to 5 can be expressed as

$$q_{5}=\frac{Aq_{1}+B}{Cq_{1}+D},$$ (7)

where A, B, C, and D are the elements of the matrix M defined in Eq. (5).

According to the properties of Gaussian beams, the radius of curvature of the wavefront at the focal plane tends to infinity, i.e., R→∞. Applying this condition to the above equations, the working distance $z_{\omega }$ can be expressed as

$$z_{\omega }=\frac{\frac{n-n_{0}}{rn_{0}}-\frac{\lambda^{2}n_{f}^{2}sL}{n^{3}\omega_{0}^{4}\pi^{2}}}{\frac{n-n_{0}}{r^{2}n_{0}^{2}}+\frac{\lambda^{2}n_{f}^{2}s^{2}}{n^{4}\omega_{0}^{4}\pi^{2}}},$$ (8)

where $\omega_{0}$ is the waist radius at plane 1 and the expression of the parameter s is given below

$$s=\frac{n}{n_{0}}-\frac{n(n-n_{0})L}{rn_{0}}.$$ (9)

The waist diameter ($2\omega_{f}$) of the focused Gaussian beam can then be determined using M and $z_{\omega }$ and expressed as

$$2\omega_{f}=2\omega_{0}\sqrt{\frac{n_{f}}{n_{0}}(\frac{A^{2}+(\lambda /n\pi \omega_{0}^{2}{)}^{2}B^{2}}{AD-BC})}.$$ (10)

It can be seen from Eqs. (8) and (10) that the working performance of the lensed fiber-based probe is dependent on the values of the parameters $n_{f}$, n, $n_{0}$, λ, $\omega_{0}$, L, and r. By selecting appropriate values for L and r, the desired working distance and spot size of the focused light beam can be achieved.

**Fig. 3:**
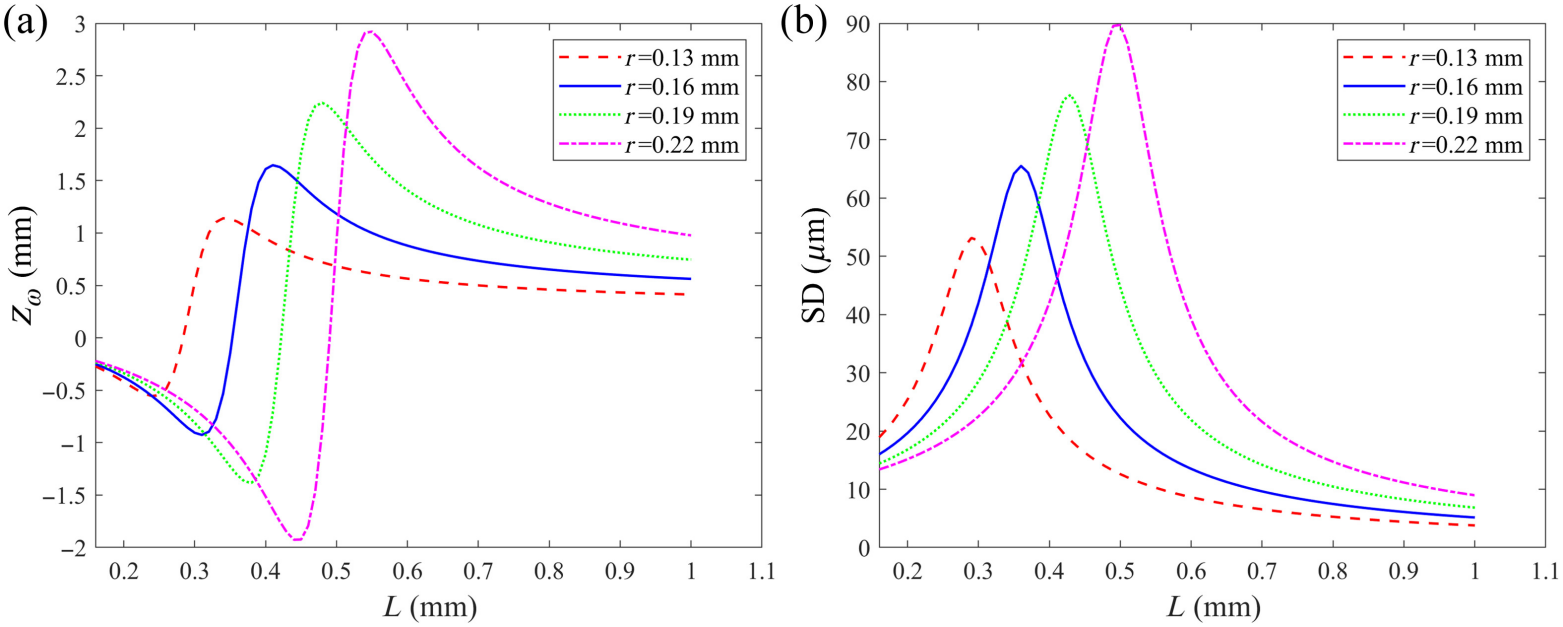
(a) Working distance and (b) the focused spot diameter of the probe dependent on the length of the NCF and the curvature radius of the ball lens.

The working distance and the focused spot diameter were quantitatively analyzed with L and r as the variable parameters. The values of $n_{f}$, n, $n_{0}$, λ, and $\omega_{0}$ are set to the typical values from the specification of the commercially available products. The working distance and focused spot diameter varied with the value of L corresponding to the different values of r, which are calculated according to Eqs. (8) and (10). The results are presented in Fig. 3.

As shown in Fig. 3(a), the working distance initially decreases with increasing L, then reaches a maximum, and finally decreases. From Fig. 3(b), the focused spot diameter first increases and then decreases with increasing L. As L and r can be independently adjusted, the probe is able to achieve the desired working distance and focused spot diameter.

According to the curves shown in Fig. 3, the L and r were selected as 0.53 and 0.16 mm, respectively. The theoretical values of the working distance and focused spot diameter were 1.1 mm and 18.68  μm, respectively.

#### 2.2.2 Fabrication and test of the probe

**Fig. 4:**
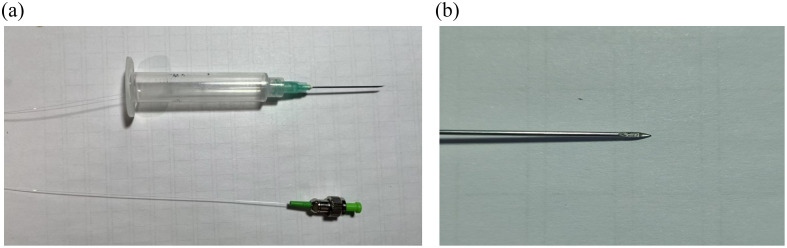
(a) Physical view of the fully assembled probe and (b) close-up of the tip of the assembled probe.

To fabricate the manual scanning OCT probe, a segment of the NCF (CL 1010-A, CFC, China) is spliced to the end of the SMF (SMF-28E, Corning, USA), and then, the fiber ball lens is fused at the end of the NCF using a fusion splicer (FSU-975, Ericsson Inc.). A 48-deg TIR surface was polished onto the ball lens using a fiber optic grinder (Nanopol, Ultra Tec Inc., USA). The completed lensed fiber assembly was inserted into a glass capillary tube, which is encapsulated in a 21-gauge needle (0.8 mm outer diameter). Finally, epoxy resin was applied to seal and protect the exposed capillary section at the needle tip. The photograph of the fully assembled manual scanning OCT probe is shown in Fig. 4(a), and the close-up of the tip of the probe is presented in Fig. 4(b).

**Fig. 5:**
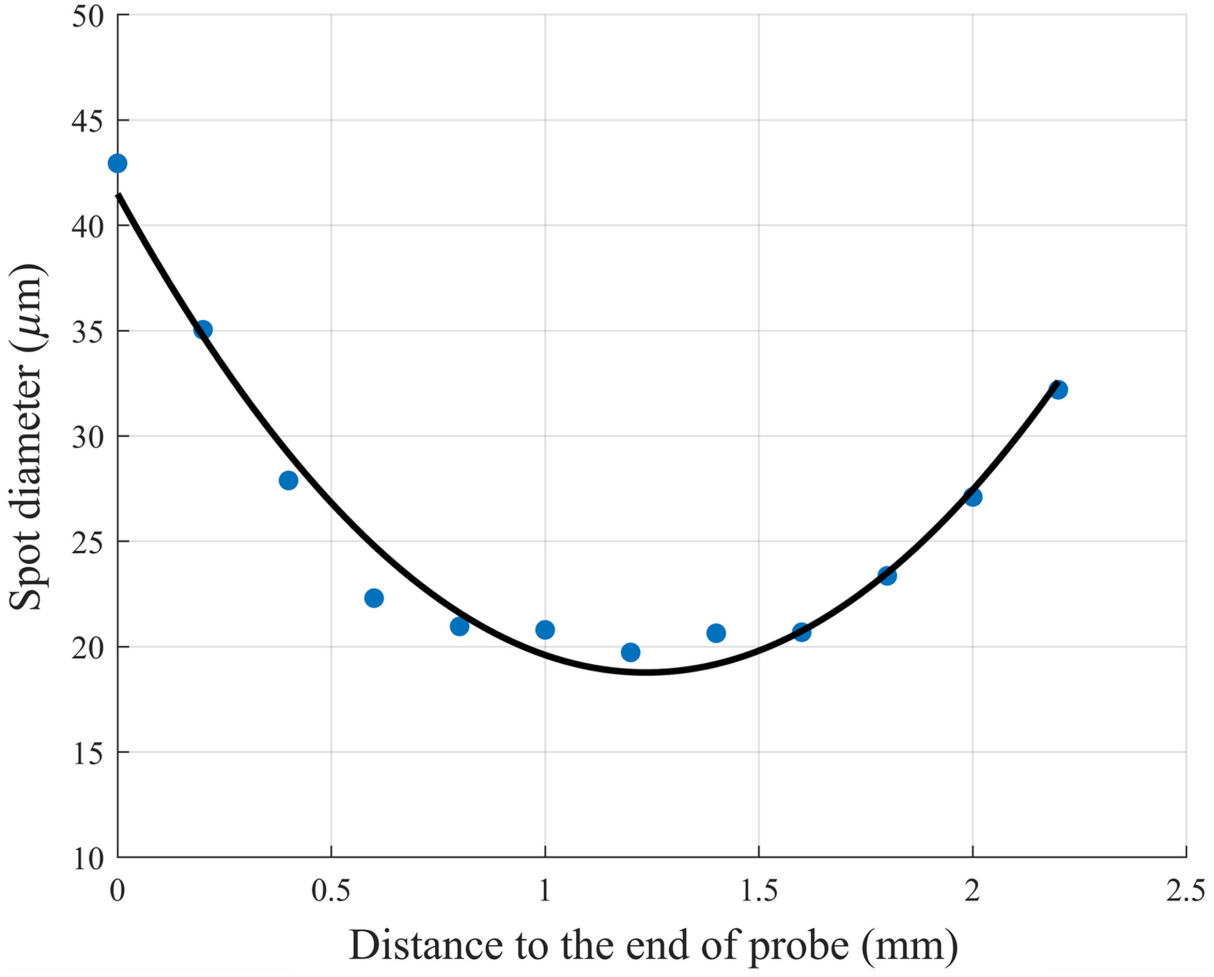
Measured spot diameter at different distances from the end of the probe.

To test the performance of the assembled probe, the spot size at different locations from the end of the probe is measured by a microscopic objective lens and an infrared CCD camera (C14041-10U, Hamamatsu). The relationship curve between the distance to the end of the probe and the spot diameter is shown in Fig. 5. The blue dots represent the measured values, and the black solid line is the fitted curve. As can be seen from Fig. 5, the spot diameter reaches the minimum value of 18.78  μm at a distance of 1.22 mm from the end of the probe, which demonstrates good agreement with the theoretical values.

### 2.3 Manual Scanning Motion Correction

The proposed manual scanning OCT needle probe utilizes the motion correction method based on speckle decorrelation, as previously described in Ref. 11. This method estimates the lateral displacement (Δx) between adjacent A-lines to correct artifacts arising from non-constant scanning speed. The cross-correlation coefficient (XCC) between adjacent A-lines can be calculated by

$$\rho_{I_{x}(z),I_{x+\Delta x}(z)}=\frac{\left\langle [I_{x}(z)-\langle I_{x}(z)\rangle ][I_{x+\Delta x}(z)-\langle I_{x+\Delta x}(z)\rangle ]\right\rangle }{\sigma_{I_{x}(z)}\sigma_{I_{x+\Delta x}(z)}},$$ (11)

where $I_{x}(z)$ and $I_{x+\Delta x}(z)$ are the OCT intensity profiles at lateral positions x and x+Δx, respectively. The symbol ⟨ ⟩ represents taking the average value of the data, and σ denotes the standard deviation.

The XCC of the fully developed speckle is solely determined by Δx. Therefore, the time-varying Δx between the adjacent A-lines can be calculated from the XCC using the relationship established in Ref. 11.

$$\Delta x=\omega_{f}\sqrt{\ln(\frac{1}{\rho_{I_{x}(z),I_{x+\Delta x}(z)}})},$$ (12)

where $\omega_{f}$ is the waist radius of the focused Gaussian beam. Consider a scan frame containing N A-lines obtained in the lateral positions $x_{1},x_{2},\ldots ,x_{N}$ during manual scanning. As can be seen from Eq. (12), the interval ($\Delta x_{i}$) between the $x_{i}$ and $x_{\left(i+1\right)}$ A-lines can be extracted using the XCC between the $I_{i}(z)$ and $I_{i+1}(z)$. Thus, the total distance between the first and last A-lines in the frame $\Delta x_{\text{total}}$ can be estimated by summing all the $\Delta x_{i}$, i.e., $\Delta x_{\text{total}}=\sum \Delta x_{i}$. With a preset sampling interval $\Delta x_{s}$, the A-line data corresponding to the uniformly distributed lateral locations $0,\Delta x_{s},2\Delta x_{s},\ldots ,M\Delta x_{s}$, can be retrieved by data interpolation, where M is the total number of the retrieved A-line, i.e., $M=\Delta x_{\text{total}}/\Delta x_{s}$.

### 2.4 Biological Sample Imaging

#### 2.4.1 Phantom imaging

**Fig. 6:**
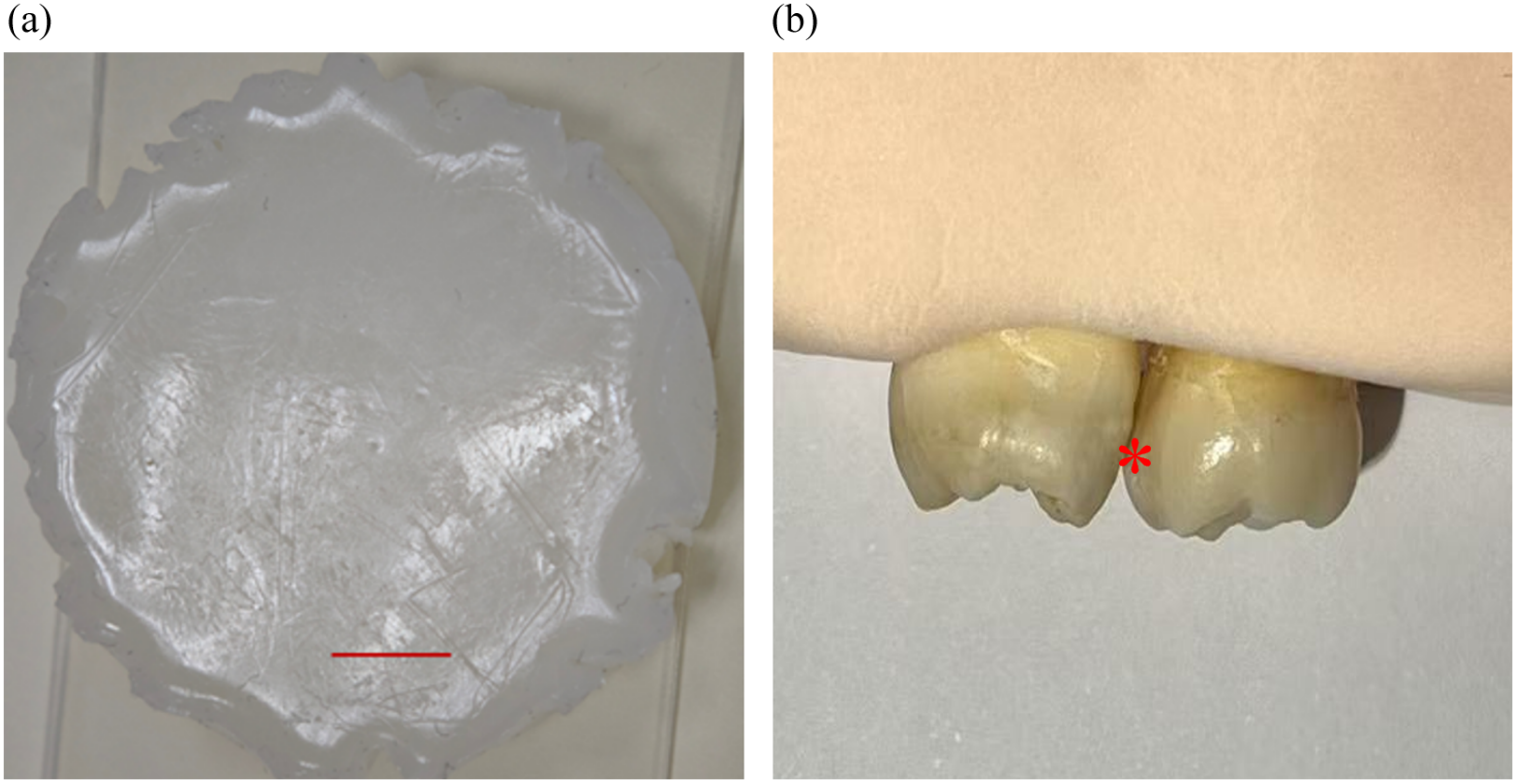
(a) Photograph of the ${\mathrm{TiO}}_{2}$ phantom. The probe was manually scanned along the red line. (b) Photograph of the prepared tooth sample and the red asterisk indicate the location of the interproximal caries.

To test the imaging performance of the manually scanned needle probe and validate the efficacy of the decorrelation algorithm for displacement extraction, a titanium dioxide (${\mathrm{TiO}}_{2}$) phantom was prepared. To fabricate the phantom, 0.4 g of ${\mathrm{TiO}}_{2}$ powder and 0.7 g of agar powder were added to a beaker containing 70 mL of water. The beaker was heated over an alcohol burner’s outer flame using the asbestos net, with constant stirring using a glass rod. After heating for several minutes, the burner was removed and the mixture was left to solidify. The resulting phantom was a highly scattering medium with the particle concentration of 0.5%, as shown in Fig. 6(a). When the phantom was imaged, the tip of the probe was manually scanned along the red line shown in Fig. 6(a), covering a range of ∼5  mm.

#### 2.4.2 Human finger skin imaging

To evaluate the imaging performance of the self-made needle probe on biological tissue, imaging experiments were performed on the healthy human finger skin as the biological sample. The operator adjusted the probe-to-skin distance to ensure the focused beam spot exited the needle opening located on the skin surface. The needle probe was then manually moved along a single direction across the skin surface to complete the scan.

#### 2.4.3 Interproximal caries imaging

To assess the probe’s capability for direct interproximal caries detection, a custom-made interproximal caries sample was prepared. Two human teeth freshly extracted were used as the sample. The blood and saliva on the surface were removed by rinsing with normal saline. To simulate the interproximal caries, a 2  mm×1  mm×1  mm cavity was prepared on the proximal surface of one tooth by using a high-speed dental handpiece with cold water spray. The two teeth were fixed in proximity using modeling clay to form a narrow interproximal space. The photograph of the teeth sample is shown in Fig. 6(b). The tooth on the left side is the healthy tooth without any cavity, whereas the right one is the tooth with the prepared cavity (indicated by the red asterisk). In the experiment, the tip of the probe was manually scanned through the interproximal space between the two teeth.

All experimental procedures were in accordance with the guidelines approved by Nanjing Medical University. Written informed consent was obtained after all procedures were fully explained to the subjects. For the extracted teeth (which constitute anonymized biological samples without personal identifiers), the requirement for individual consent was waived by the oversight body in accordance with institutional guidelines and applicable regulations.

## 3 Results

**Fig. 7:**
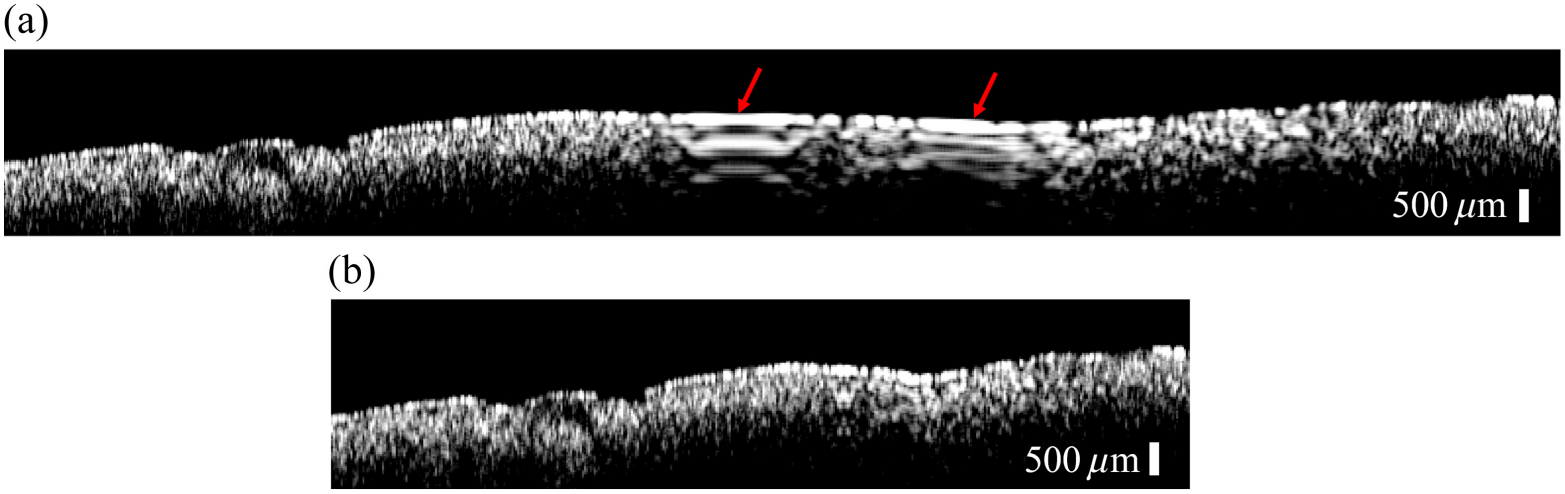
Reconstructed OCT images without (a) and with (b) the scanning speed correction. Red arrows in panel (a) indicate the motion artifacts.

The raw OCT image of the ${\mathrm{TiO}}_{2}$ phantom is shown in Fig. 7(a), which consists of 5000 A-lines. The motion artifacts are indicated by the red arrows. Application of the decorrelation algorithm with the sampling interval of 5.5  μm yielded the artifact-free reconstructed image. The reconstructed image contains 2586 A-lines, as shown in Fig. 7(b). This result demonstrates that the decorrelation algorithm can be used to correct the motion artifacts induced by the non-uniform manual scanning velocities.

**Fig. 8:**
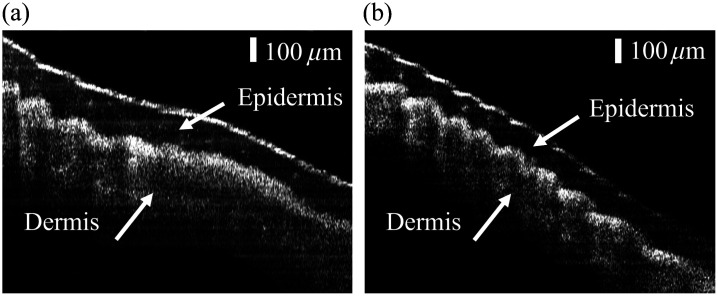
Reconstructed OCT images of the skin tissue of the middle finger (a) and index finger (b) obtained using the manual scanning OCT probe.

In the finger skin imaging experiment, the probe was manually scanned across the surfaces of the index finger and middle finger, respectively. A total of 5000 A-lines were acquired and processed using the decorrelation algorithm. Figures 8(a) and 8(b) present two reconstructed artifact-free OCT images of the skin tissue, which contain 3763 and 3661 A-lines, respectively. The epidermal-dermal junction is clearly revealed in the images. This demonstrates that the needle probe is capable of resolving biological structures while compensating for the motion artifacts caused by the non-uniform manual scanning speeds.

**Fig. 9:**
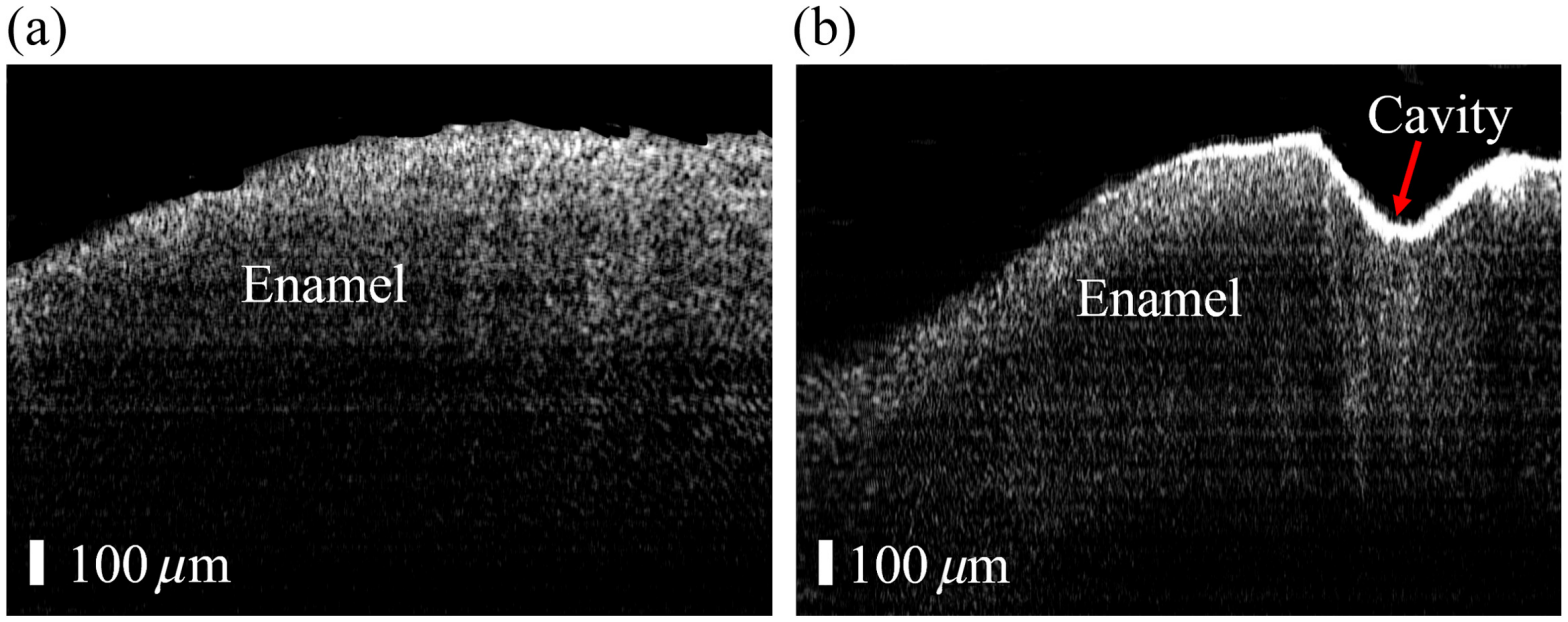
Reconstructed manually scanned OCT images of the healthy tooth (a) and the tooth with the interproximal caries (b).

The imaging performance of the probe was further validated by imaging the interproximal caries directly. The reconstructed artifact-free OCT image of the intact tooth is shown in Fig. 9(a), which contains 3775 A-lines. From the figure, the intact enamel structure of the left tooth can be seen clearly, and no cavity is present on the proximal surface. The reconstructed artifact-free image of the defective tooth is shown in Fig. 9(b), which contains 3984 A-lines. A distinct interproximal cavity is seen on the proximal surface of the right tooth (red arrow), despite preserved enamel architecture. This direct imaging of the interproximal caries demonstrates the ability of the developed manual scanning OCT needle probe for high-sensitivity diagnosis of the interproximal caries.

## 4 Discussion

Although the proposed ultrathin lensed fiber-based needle probe enables the direct imaging of the interproximal caries via manual scanning, certain limitations persist in the clinical implementation. One of the limitations is that the speed and trajectory of the manual scanning are inevitably subject to the operator-induced variations. Although the decorrelation algorithm effectively corrects the motion artifacts under the laboratory conditions, hand tremor and confined intraoral space may compromise the image reproducibility *in vivo*. Further improvement may consider integrating certain motion stabilization mechanisms (e.g., inertial sensors) to stabilize the manual scanning. The other limitation is that it is difficult to standardize the matrix array of all reconstructed images. With the decorrelation algorithm, the number of A-lines in the reconstructed image is determined by the equation $M=\Delta x_{\text{total}}/\Delta x_{s}$, where $\Delta x_{\text{total}}$ is the total distance from the first to the last A-line in the raw image, and $\Delta x_{s}$ is the preset evenly-spaced sampling interval. The value of $\Delta x_{\text{total}}$ varies for each manual scanning. The value of $\Delta x_{s}$, which is determined by the lateral displacement between the two adjacent A-lines in the region of uniform motion, is also an unpredictable variable. Thus, the values of $\Delta x_{\text{total}}$ and $\Delta x_{s}$ are both independent variables, and it is hard to directly obtain the images with the same array size. However, the image interpolation algorithm can be further used to standardize the array size for better comparison of different images. Due to the birefringent properties of teeth, it will be possible in the future to combine the needle probe with the polarization-sensitive OCT technology to obtain the polarization properties of the teeth tissue. The proposed needle probe may enable more advanced clinical applications in the future, such as distinguishing the early-stage of the caries (white spot lesion) and assessing the lesion activity.

## 5 Conclusion

In summary, this study proposed an ultrathin lensed fiber-based manual scanning OCT needle probe. The experimentally measured working distance and focused spot diameter of the probe were 1.22 mm and 18.78  μm, respectively. By applying the decorrelation algorithm for the manual scanning image reconstruction, motion artifacts were effectively eliminated, yielding high-quality OCT images. Critically, the probe enables the direct imaging of the interproximal surface between two adjacent teeth, clearly distinguishing the structural differences between the healthy tooth tissue and the cavitated interproximal caries. These results comprehensively validate the efficacy of the proposed ultrathin needle probe and its potential for detecting interproximal caries, offering a promising novel diagnostic tool for future clinical applications.

## Data Availability

Data and code developed in this study are available upon reasonable request to the corresponding author.
